# Molecular epidemiology and genetic diversity of norovirus among hospitalized children with acute gastroenteritis in Tianjin, China, 2018–2020

**DOI:** 10.1186/s12879-021-06375-2

**Published:** 2021-07-14

**Authors:** Yulian Fang, Zhaoying Dong, Yan Liu, Wei Wang, Mengzhu Hou, Jinying Wu, Lu Wang, Yu Zhao

**Affiliations:** 1 Tianjin Pediatric Research Institute, Tianjin Children’s Hospital (Children’s Hospital of Tianjin University), Tianjin Key Laboratory of Birth Defects for Prevention and Treatment, No.238 Longyan Road, Beichen District, Tianjin, 300134 China; 2 Department of Neurology, Tianjin Union Medical Centre, No. 190 Jieyuan Road, Hongqiao District, Tianjin, 300121 China; 3 Clinical Pediatric College of Tianjin Medical University, No.238 Longyan Road, Beichen District, Tianjin, 300134 China; 4 Department of Digestion, Tianjin Children’s Hospital (Children’s Hospital of Tianjin University), No.238 Longyan Road, Beichen District, Tianjin, 300134 China

**Keywords:** Norovirus, Acute gastroenteritis, Genotype, RdRp, Capsid, Children

## Abstract

**Background:**

Norovirus (NoV) is a major cause of viral acute gastroenteritis (AGE) in children worldwide. Epidemiological analysis with respect to the virus strains is limited in China. This study aimed to investigate the prevalence, patterns, and molecular characteristics of NoV infection among children with AGE in China.

**Methods:**

A total 4848 stool samples were collected from children who were admitted with AGE in Tianjin Children’s Hospital from August 2018 to July 2020. NoV was preliminarily detected using real-time reverse transcription polymerase chain reaction (RT-PCR). Partial sequences of the RNA-dependent RNA polymerase (RdRp) and capsid genes of positive samples were amplified by conventional RT-PCR and then sequenced. The NoV genotype was determined by online Norovirus Typing Tool Version 2.0, and phylogenetic analysis was conducted using MEGA 6.0.

**Results:**

The prevalence of NoV was 26.4% (1280/4848). NoV was detected in all age groups, with the 7–12 months group having the highest detection rate (655/2014, 32.5%). NoV was detected during most part of the year with higher frequency in winter than other seasons. Based on the genetic analysis of RdRp, GII. Pe was the most predominant genotype detected at 70.7% (381/539) followed by GII.P12 at 25.4% (137/539). GII.4 was the most predominant capsid genotype detected at 65.3% (338/518) followed by GII.3 at 26.8% (139/518). Based on the genetic analysis of RdRp and capsid sequences, the strains were clustered into 10 RdRp–capsid genotypes: GII.Pe-GII.4 Sydney 2012 (65.5%), GII.P12-GII.3 (27.2%), GII.P16-GII.2 (1.8%), GII.P12-GII.2 (0.2%), GII.P17-GII.17 (1.1%), GII.Pe-GII.3 (1.8%), GII.Pe-GII.2 (1.1%), GII.Pe-GII.1 (0.4%), GII.16-GII.4 Sydney 2012 (0.7%), and GII.P7-GII.6 (0.2%). The predominant NoV genotypes changed from GII.Pe-GII.4 Sydney 2012 and GII.P12-GII.3 between August 2018 and July 2019 to GII.Pe-GII.4 Sydney 2012 and GII.P16-GII.2 between August 2019 and July 2020. The patients with GII.Pe-GII.4 Sydney 2012 genotype were more likely to suffer from vomiting symptom than those with GII.P12-GII.3.

**Conclusions:**

NoV is an important pathogen responsible for viral AGE among children in China. GII.Pe-GII.4 Sydney 2012 and GII.P12-GII.3 were major recombinant genotypes. Knowledge of circulating genotypes and seasonal trends is of great importance for disease prevention and surveillance.

## Background

Norovirus (NoV) is a leading cause of epidemic and sporadic acute gastroenteritis (AGE) in people of all ages worldwide. Its symptoms include nausea, vomiting, diarrhea, abdominal pain, dehydration, and fever [1]. While these symptoms can be self-limiting among healthy people, elderly and immunocompromised individuals often develop severe illness [2]. NoV infections are of concern given the significant burden it places on public health, particularly in hospital, schools, and aged-care facilities. Currently, there are no effective treatment methods for NoV infections, and vaccines are in late development stages [3].

NoV belongs to the *Caliciviridae* family, and the genome is approximately 7.5 kb in length and contains three open reading frames (ORF). ORF-1 encodes six non-structural proteins that include the RNA-dependent RNA polymerase (RdRp); ORF-2 and ORF-3 encode the major capsid protein (VP1) and minor structural protein (VP2), respectively [4]. NoV is divided into 10 genogroups, of which GI and GII are responsible for the disease in humans. Capsid protein VP1 and RdRp sequences are the basis of current genetic classification of NoV [5]. To date, 9 and 27 capsid genotypes and 14 and 37 RdRp genotypes have been reported for GI and GII, respectively [6]. Routinely, the NoV genotype was determined by only RdRp (ORF1) or VP1 (ORF2) gene sequence. Until recent years, more and more researchers found that NoV often undergoes recombination at the ORF1–ORF2 junction, which leads to a single virus strain clustering under different genotypes when different genomic regions are used for sequencing [7, 8]. Moreover, the recombination occurs more often than previously recognized [9]. Thus, genotyping of NoV should ideally be based on sequencing of the capsid and RdRp.

Epidemiologic studies have shown that most human NoV infections are caused by GII genotype, of which GII.4 is responsible for the majority of outbreaks and sporadic cases worldwide [10]. Interestingly, new GII.4 variants have emerged and rapidly replace the dominant circulating strain every 2–3 years [11]. Related studies have confirmed that GII.4 NoV evolution is driven by antigenic drift and recombination [12]. At present, the recombinant NoV genotypes GII.Pe-GII.4 Sydney 2012 and GII.P12-GII.3 are the predominant strains in outbreaks of AGE in many cities of China [13], which could help improve our understanding of the molecular epidemiology of NoV.

The present study aimed to evaluate the changing pattern of prevalence and genetic diversity of NoV among children with AGE in Tianjin. This study not only described the epidemiological features and diversity of circulating NoV, but also compared the clinical manifestations among children with different genotypes. Epidemiological knowledge of NoV and their evolution over the years is critical for the continued development of effective preventive measures and treatment.

## Methods

### Sample collection

A total of 4848 stool specimens were collected from children who had been hospitalized with AGE at Tianjin Children’s Hospital from August 2018 to July 2020. Stool samples were collected from patients admitted with AGE. AGE was defined as follows: diarrhea (loose/watery stool) or vomiting with a frequency of three or more times in 24 h [14]. NoV-associated AGE was further defined by the above clinical symptoms and laboratory confirmatiom of NoV. The age of participants ranged from 1 month to 148 months. All stool specimens were stored at − 80 °C. The study was conducted according to the Helsinki guidelines and approved by the ethics committee of Tianjin Children’s Hospital.

### NoV RNA isolation and detection

Stool samples were diluted to 10% suspension with phosphate-buffered saline and then clarified by centrifugation at 3000×g for 10 min. Viral RNA was extracted using the QIAamp Viral RNA Mini Kit (Qiagen, Heiden, Germany) according to the manufacturer’s instructions and stored at − 80 °C. Then, Real-Time RT-PCR for NoV detection was carried out using Norovirus Real-Time RT-PCR kit (Langde, China) on a 7500 Real Time PCR platform according to the manufacturer’s instructions. The final volume for each reaction was 50 μL. Each reaction contained 35.8 μL of reaction mixture, 4.2 μL of enzyme mix, and 10 μL of RNA. The amplification conditions were set as follows: reverse transcription 42 °C for 30 min, followed by 95 °C for 3 min, then 40 cycles of 95 °C for 10 s, 60 °C for 1 min. Targets with cycle threshold values ≤36 were considered positive for that particular sample according to results interpretation of the kit. All samples were not screened for other viruses.

### NoV RdRp and capsid amplification

In an effort to identify the NoV genotype, positive samples were analyzed by conventional real-time reverse transcription polymerase chain reaction (RT-PCR) using the TaKaRa Ex Taq Hot Start RT-PCR Kit (TaKaRa Shuzou, Kyoto, Japan). The RdRp gene was amplified using the JV12Y/JV12I primer set for RdRp typing [15]. The capsid gene was amplified using the G1SKF/R and G2SKF/R primers for capsid typing for GI and GII, respectively [16]. Each 25 μL reaction mixture contained 2.5 μL of 10× Ex Taq buffer, 2 μL of dNTPs (2.5 mM), 1 μL of each primer, 0.25 μL of Taq DNA polymerase (5 U/μL), and 5 μL of cDNA and added with 13.25 μL of ddH_2_O to reach a volume of 25 μL. PCR was performed under the following conditions: 94 °C for 5 min followed by 35 cycles, 94 °C for 3 min, 55 °C for 45 s, 72 °C for 1 min, and final extension at 72 °C for 7 min. All RT-PCR products were analyzed by 1.5% agarose gel electrophoresis. The PCR products were purified and sent to the GENEWIZ Company for sequencing. Then, the genotypes of all NoV strains sequenced successfully were determined by nucleotide sequence analysis using the online Norovirus Typing Tool Version 2.0 (https://www.rivm.nl/mpf/typingtool/norovirus/).

### Phylogenetic analysis

The phylogenetic relationships of NoV were analyzed by aligning sequences using ClustalW software and Kimura’s two parameters. The phylogenetic trees of nucleotide sequences of RdRp and capsid genes were constructed using the neighbor-joining method by MEGA Version 6.0 software and validated by 1000 bootstrap replicates [17].

### Statistical analysis

All data were analyzed using SPSS software version 19.0. Categorical and continuous variables were compared by Chi-square test and *t*-test, respectively. *P* values < 0.05 were considered statistically significant.

## Results

### Clinical and epidemiological data

**Fig. 1:**
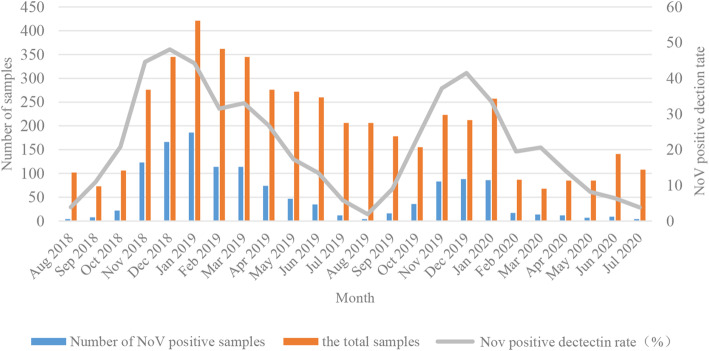
Monthly distribution of NoV infection in chidlren with AGE in Tianjin from 2018 to 2020

Among the 4848 children with AGE enrolled in the study, 2956 (61.0%) were male, and 1889 (39.0%) were female. NoV was detected at a higher rate in males than in females, but the difference was not statistically different (*P* = 0.701). NoV was detected in all age groups tested (< 6, 7–12, 13–36, 37–84, ≥85) with the highest detection rate in the 7–12 months group (32.5%, 655/2014), followed by the 13–36 months group (26.9%, 235/874), 37–84 months group (21.3%, 105/494), ≥85 months group (20.9%, 49/235), and 0–6 months group (19.2%, 236/1231). The difference was statistically significant (*P* = 0.000). According to the characteristics of the climate in China, 1 year is divided into four seasons, including spring (March–May), summer (June–August), autumn (September–November), and winter (December–February). Then, the seasonal pattern of NoV was analyzed. NoV infection was identified in all seasons. A significant difference was observed in the NoV detection rate across seasons (*P* = 0.000), and the highest NoV detection rate was recorded in winter (39.0%, 657/1684), followed by autumn (28.4%, 287/1010), spring (23.7%, 268/1131), and summer (6.6%, 68/1023). The clinical and epidemiology data are shown in Table 1 and Fig. 1.

**Table 1:** Demographic information and clinical characteristics of NoV infection in children with AGE in Tianjin from 2018 to 2020

	Overall (*n* = 4848)	NoV-postive (*n* = 1280)	*χ*^*2*^	*P* value
Gender				
Male	2959	787	0.147	0.701
Female	1889	493		
Age (months)				
0 ~ 6	1231	236		
7 ~ 12	2014	655		
13 ~ 36	874	235	82.512	**0.000**
37 ~ 84	494	105		
≥ 85	235	49		
Season				
Spring	1131	268		
Summer	1023	68		
Autumn	1010	287	349.678	**0.000**
Winter	1684	657

### NoV prevalence and distribution of genogroups and genotypes

A total of 4848 subjects were enrolled in the primary study and provided stool specimens. About 1280 (26.4%) subjects were positive for NoV by real-time RT-PCR. NoV-positive samples were further genotyped, and the genotype of the capsid and/or RdRp was obtained in 608 samples.

**Fig. 2:**
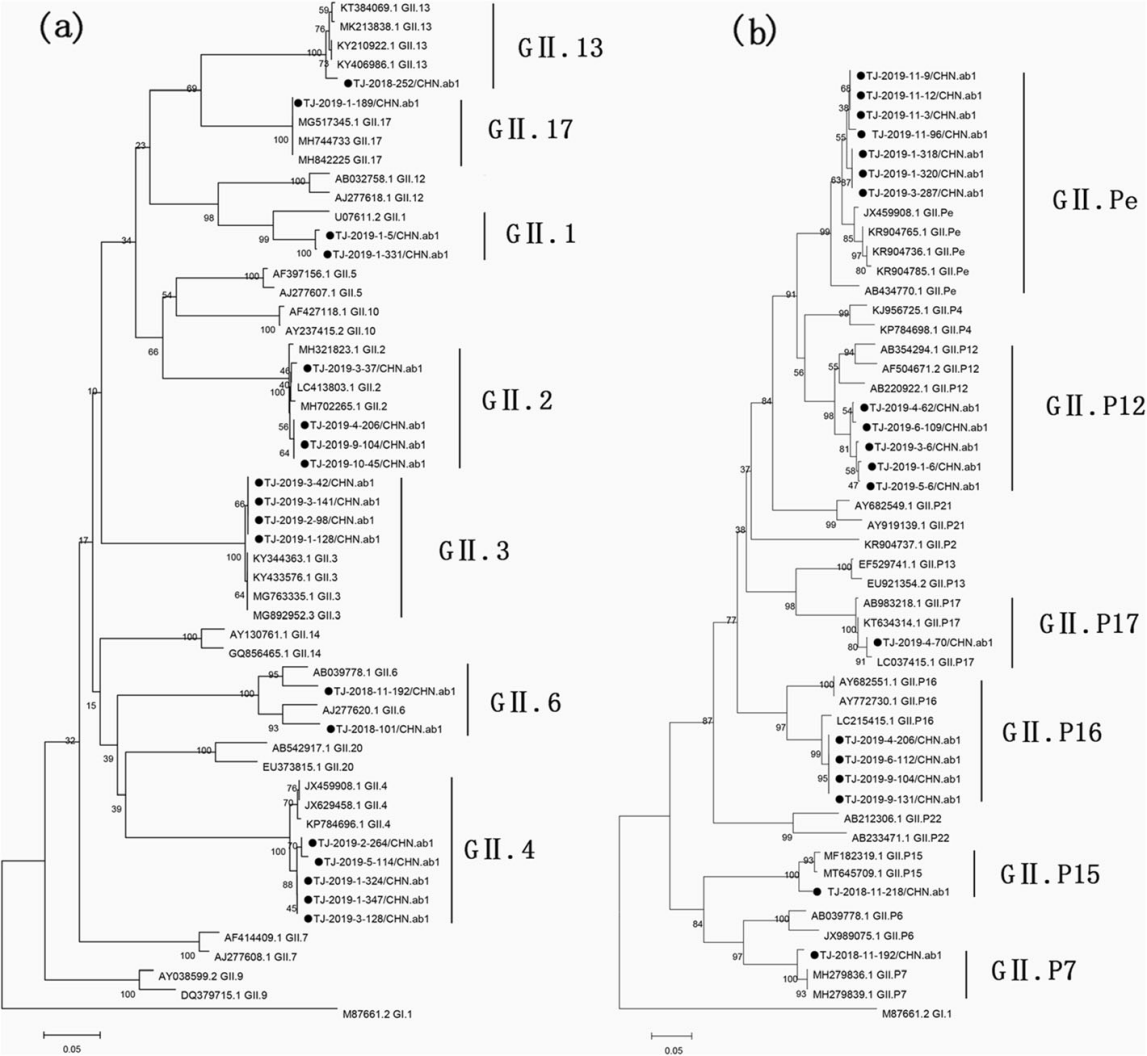
Phylogenetic tree of NoV based on partial capsid (**А**) and RdRp (**B**) genes of NoV strains detected in children with AGE in Tianjin, 2018–2020. The study samples are in shaded circles and unmarked reference strains are indicated by GenBank accession numbers. The scale bar represents nucleotide substitutions per site and the number above each branch corresponds to the bootstrap value. Scale bar is proportional to genetic distance

The genotype was obtained from two genes (RdRp and capsid) in 449 specimens (73.8%, 449/608), from the RdRp gene alone in 90 specimens (14.8%, 90/608), and from the capsid gene alone in 69 specimens (11.3%, 69/608). In total, six RdRp-based (539 samples) and seven capsid-based (518 samples) genotypes were identified in the GII genogroup as shown by the constructed phylogenetic tree in Figs. 2A and B, respectively. Based on the capsid gene, the GII.4 strain was predominant with 65.3% (338/518); other strains identified were GII.3 at 26.9% (139/518), GII.2 at 5.2% (27/518), GII.1 at 1.2% (6/518), GII.17 at 1.0% (5/518), GII.6 at 0.4% (2/518), and GII.13 at 0.2% (1/518). The predominant NoV genotypes changed from GII.4 Sydney 2012 and GII.3 between August 2018 and July 2019 to GII.4 Sydney 2012 and GII.2 between August 2019 and July 2020. Regarding classification by the RdRp gene, GII. Pe was the most prevalent at 70.7% (381/539), followed by GII.P12 at 25.4% (137/539), GII.P16 at 2.2% (12/539), GII.P17 at 1.1% (6/539), GII.P7 at 0.4% (2/539), and GII.P15 at 0.2% (1/347). The most common genotypes changed from GII. Pe and GII.P12 between August 2018 and July 2019 to GII. Pe and GII.P16 between August 2019 and July 2020 (Table 2).

**Table 2:** Genotype distribution of NoV positive samples among children with AGE in Tianjin from 2018 to 2020

Gentype	Number (n, %)		Total Number (n, %)
	2018 ~ 2019	2019 ~ 2020	
RdRp			
GII.P12	135 (34.1)	2 (1.4)	137 (25.4)
GII.P15	1 (0.3)	0	1 (0.2)
GII.P16	5 (1.3)	7 (4.9)	12 (2.2)
GII.P17	6 (1.5)	0	6 (1.1)
GII.P7	1 (0.3)	1 (0.7)	2 (0.4)
GII.Pe	248 (62.6)	133 (93.0)	381 (70.7)
Capsid			
GII.1	6 (1.6)	0	6 (1.2)
GII.13	1 (0.3)	0	1 (0.2)
GII.17	5 (1.3)	0	5 (1)
GII.2	17 (4.5)	10 (7.2)	27 (5.2)
GII.3	137 (36.1)	2 (1.4)	139 (26.8)
GII.6	2 (0.5)	0	2 (0.4)
GII.4 Sydney 2012	211 (55.7)	127 (91.4)	338 (65.3)
RdRp/Capsid			
GII.Pe-GII.4 Sydney 2012	190 (56.9)	104 (90.4)	294 (65.5)
GII.P12-GII.3	120 (35.9)	2 (1.7)	122 (27.2)
GII.P16-GII.2	4 (1.2)	4 (3.5)	8 (1.8)
GII.P12-GII.2	1 (0.3)	0	1 (0.2)
GII.Pe-GII.3	8 (2.4)	0	8 (1.8)
GII.Pe-GII.2	3 (0.9)	2 (1.7)	5 (1.1)
GII.Pe-GII.1	2 (0.6)	0	2 (0.4)
GII.P17-GII.17	5 (1.5)	0	5 (1.1)
GII.P7-GII.6	1 (0.3)	0	1 (0.2)
GII.P16-GII.4 Sydney 2012	0	3 (2.6)	3 (0.7)

Combined RdRp and capsid genotypes of 449 strains were determined, resulting in 10 possible variants, including GII.Pe-GII.4 Sydney 2012, GII.P12-GII.3, GII.P16-GII.2, GII.P12-GII.2, GII.Pe-GII.3, GII.Pe-GII.2, GII.Pe-GII.1, GII.P17-GII.17, GII.P7-GII.6, and GII.P16-GII. Sydney 2012. The most common recombinant RdRp–capsid genotype was GII.Pe-GII.4 Sydney 2012 at 65.5%, followed by GII.P12-GII.3 at 27.2%, GII.P16-GII.2 and GII.Pe-GII.3 each at 1.8%, GII.P17-GII.17 and GII.Pe-GII.2 each at 1.1%, GII.16-GII.4 Sydney 2012 at 0.7%, GII.Pe-GII.1 at 0.4%, and GII.P12-GII.2 and GII.P7-GII.6 each at 0.2%. The results for NoV genotype of RdRp and capsid genes are shown in Table 2. Discordant RdRp and capsid genotypes were identified in 444 of 449 genotypes, including GII.Pe-GII.4 Sydney 2012 (*n* = 294), GII.P12-GII.3 (*n* = 122), GII.P16-GII.2 (*n* = 8), GII.P12-GII.2 (*n* = 1), GII.Pe-GII.3 (*n* = 8), GII.Pe-GII.2 (*n* = 5), GII.Pe-GII.1 (*n* = 2), GII.P7-GII.6 (*n* = 1), and GII.P16-GII. Sydney 2012 (*n* = 3), which suggested the presence of intergenotype recombinant strains, although it needs to be confirmed.

**Fig. 3:**
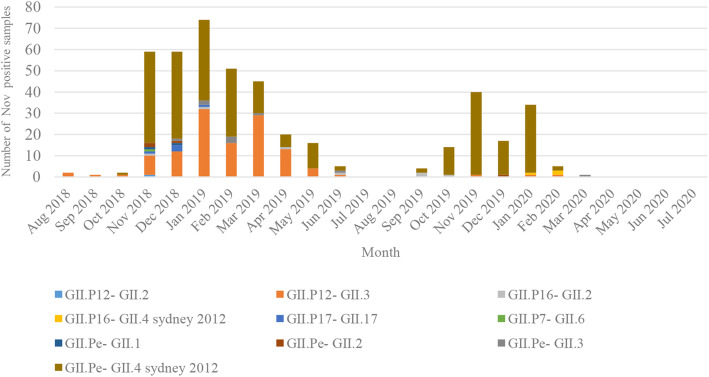
Monthly distribution of NoV genotypes among children with AGE in Tianjin from 2018 to 2020

The predominant genotypes of NoV during our study period are diverse according to month. The number of NoV genotype in each month is shown in Fig. 3. The predominant NoV genotype was GII.Pe-GII.4 Sydney 2012 in August 2018 and July 2019, accounting for 56.9% (190/334) of genotyped strains, followed by GII.P12-GII.3 (35.9%, 120/334). Interestingly, GII.Pe-GII.4 Sydney 2012 was still the predominant genotype in August 2019 and July 2020, accounting for 90.4%, followed by GII.P16-GII.2, accounting for 3.5% (4/115).

### Clinical features of GII.P12-GII.3 and GII.Pe-GII.4 Sydney 2012 genotypes

The clinical features of NoV that were detected in AGE patients included diarrhea, vomiting, abdominal pain, dehydration and fever. Among 1280 patients infected with NoV, 68.2% (873/1280) reported diarrhea, 48.5% (621/1280) vomiting, 37.7% (483/1280) diarrhea and vomiting, 4.7% (60/1280) abdominal pain, 3.2% (41/1280) dehydration, and 69.6% (891/1280) fever. Among 891 patients with fever, 113 experienced mild fever (37.3 °C – 38.0 °C), 342 patients experienced moderate fever (38.1 °C – 39.0 °C), and 436 patients experienced severe fever. We also compared the clinical characteristics of NoV infection caused by GII.P12-GII.3 and GII.Pe-GII.4 Sydney 2012 in the AGE patients (Table 3). No significant differences were found in terms of clinical features except for vomiting. Patients with GII.Pe-GII.4 Sydney 2012 were more likely to suffer from vomiting (*P* = 0.000).

**Table 3:** Clinical characteristics of NoV infection caused by GII.P12-GII.3 and GII.Pe-GII.4 Sydney 2012 in the children with AGE

Parameter	GII.P12-GII.3 (n, %)	GII.Pe-GII.4 Sydney 2012 (n, %)	χ^2^ value	*P* value
Fever (> 38 °C)				
Yes	77 (63.1)	174 (59.2)		
No	45 (36.9)	120 (40.8)	**0.557**	0.456
Vomiting				
Yes	48 (39.3)	**175 (59.5)**		
No	74 (60.7)	119 (40.5)	**14.118**	**0.000**
Vomitory (times/day)				
1–2	27	82		
3–4	10	34	2.269	0.322
≥ 5	11	59		
Watery stool				
Yes	76 (62.3)	195 (66.3)		
No	46 (37.7)	99 (33.7)	0.617	0.432
Diarrheal (times/day)				
1–2	46	110		
3–4	14	46	0.851	0.654
≥ 5	16	39		
Abdominal pain				
Yes	2 (1.6)	15 (5.1)		
No	120 (98.4)	279 (94.9)	2.638	0.104
Cough				
Yes	77 (63.1)	151 (51.4)		
No	45 (36.9)	143 (48.6)	4.809	0.028
Dehydration				
Yes	0 (0)	11 (3.7)		
No	122 (100)	283 (96.3)	3.348	0.067

## Discussion

AGE remains a leading infectious cause of morbidity and mortality in developing countries. Interestingly, with the decline of rotavirus-associated diarrhea in some countries because of the introduction of the rotavirus vaccine, a large number of diarrhea cases are still seen. NoV will likely replace rotavirus as the leading cause of AGE in children [18]. Therefore, this study aimed at evaluating the prevalence and molecular epidemiology of NoV infection among hospitalized children with AGE in Tianjin from 2018 to 2020.

In this study, we analyzed NoV-associated sporadic AGE samples in Tianjin from August 2018 to July 2020. The prevalence of NoV was 26.4% in our study, which was similar to the reported data from other epidemiological studies [19]. NoV incidence was lower in 2020 than in 2018 and 2019 (*P* = 0.000). This might be related to the Covid-19 pandemic. Because people took longer at home and paid more attention to hygiene and so on during this period. Besides, students were asked to take online classes at home to reduce crowding. So, people had less opportunities for exposure to the NoV, which might be main reason for the low incidence of NoV during the Covid-19 pandemic. NoV was detected in all age groups of children with AGE, but the highest incidence of NoV was found in the 7–12 months group, consistent with some other studies [20]. This is because this age group represents the period where children begin to interact more actively with their external environment and could be easily exposed to NoV [21, 22]. Moreover, the number of NoV-positive patients with age < 36 months accounted for 88.9% of all age groups, consistent with previously reported prevalence rates [23]. Although NoV infection occurred throughout the year, the detection rate of NoV was higher in winter compared with other seasons. At the same time, related studies have shown that NoV is generally detected during cool seasons [24]. The results suggested that there was obvious seasonal difference to NoV infection, which might be due to the fact that dry and cold climates are favorable for NoV activity [25].

Taken together with the findings of this study, most genogroup II NoV strains detected in this study belong to GII.4 and GII.3 (65.3 and 26.8%, respectively), which were the most prevalent strains in children with sporadic AGE worldwide [26, 27]. Our study suggested that the GII.4 Sydney 2012 variant was the most predominant NoV variant, consistent with the worldwide epidemic [28]. Other GII genotypes were only detected at a low rate in Tianjin. Naturally occurring recombination events were common in NoV, and the most common recombination site was the ORF1–ORF2 junction localized upstream of the capsid gene. To gain a better understanding of genetic varitation, it is essential to analyze the volutionary mechanism of NoV. Several studies have investigated the volutionary pattern of NoV, especially using partial regions or complete genomes of NoV strains [29, 30]. Recombination is a major driving force of viral evolution [31], which results in a substantial exchange of genetic material. However, multiple studies indicated that the role of recombination in shaping NoV evolutionary history is largely unknown [32, 33]. Subsequently, some studies found that new RdRp may lead to a faster mutation rate and increased genetic diversity [34]. Besides, because of the different sensitivity of PCR to different regions of NoV, some samples were positive only in ORF1 or ORF2 gene, and it is difficult to determine whether they were recombination strains or not. In our study, the NoV strains circulating in Tianjin exhibited considerable genetic diversity during the study period, and at least 10 genotypes were identified based on both RdRp and capsid genes, most of which were recombinant strains, including GII.Pe-GII.4 Sydney 2012, GII.P12-GII.3, GII.P16-GII.2, GII.P12-GII.2, GII.Pe-GII.3, GII.Pe-GII.2, GII.Pe-GII.1, GII.P7-GII.6, and GII.P16-GII.Sydney 2012. The proportion of recombination strains was very high (98.9%, 444/449). We found that 65.5% of recombination strains were GII.Pe-GII.4 Sydney 2012, followed by GII.12-GII.3 (27.2%, 122/449), which is in agreement with relevant reports in China and abroad [13, 35]. Up to now, it was not found the effect of Covid-19 pandemic on diversity of NoV in 2020. Moreover, the predominant NoV genotypes changed from GII.12-GII.3 and GII.Pe-GII.4 Sydney 2012 between August 2018 and July 2019 to GII.16-GII.2 and GII.Pe-GII.4 Sydney 2012 between August 2019 and July 2020. The recombinant GII.12-GII.3 strain is a major cause of pediatric disease worldwide [36]. However, this strain appeared to be restricted to a specific geographic region, including China, Japan, and South Korea [37]. The Chinese CDC reported that the prevalence of GII.12-GII.3 was second to GII.42006b and recombinant in several provinces [19]. GII.12-GII.3 strains ranked only second to the GII.4 recombination strains. Therefore, persistent surveillance of GII.12-GII.3-associated diarrhea should be highlighted in this region.

In this study, children infected with NoV suffered from most of the common clinical symptoms of AGE, such as fever (69.6%), diarrhea (68.2%), vomiting (48.5%), abdominal pain (4.7%), and dehydration (3.2%). The symptoms showed no significant differences between patients with genotype GII.12-GII.3 and GII.Pe-GII.4 Sydney 2012, except for vomiting. Patients with GII.Pe-GII.4 Sydney 2012 genotype were more likely to suffer from vomiting compared with those with GII.12-GII.3. Therefore, we should pay more attention to the clinical symptoms of patients with GII.Pe-GII.4 Sydney 2012 genotype in the process of diagnosis and treatment. More clinical studies are needed to demonstrate whether the different clinical characteristics are associated with infection with different NoV genotypes.

## Conclusions

In conclusion, the present study indicated the genetic diversity of GII NoV circulating in Tianjin from 2018 to 2020. Similar to findings worldwide, GII.Pe-GII.4 Sydney 2012 was found to be the predominant NoV genotype in our study. However, recombinant GII.12-GII.3 strains are considered as one of the significant pediatric pathogens, highlighting the role of recombination in the evolution of NoV. The data here highlighted the importance of combination strains based on RdRp and capsid genes, and more in-depth studies of NoV epidemiology should be extended to more regions of Tianjin to better assess the prevalence of NoV strains responsible for AGE. More importantly, to gain a better understanding of genetic variation, it is essential to analyze complete genomes of NoV strains, which is crucial for understanding of the viral evolutionary mechanism. So, further study of obtaining complete genomes was conducted to get a better way to prevent and mitigate NoV-AGE.

## Data Availability

The data generated or analysed during this study are included in this pubilished article. The sequences generated have been submitted to GeneBank with accession numbers MW8220022 to MW8220024, MW866524 to MW866528, MW866532 to MW866533, MW866536 to MW866537, MW866543 to MW866545, MW866554 to MW866556 and MW866541 (https://www.ncbi.nlm.nih.gov/nuccore).

## Abbreviations

AGE: Acute gastroenteritis
NoV: Norovirus
ORFs: Open reading frames
RdRp: RAN-dependent RNA polymerase
RT-PCR: reverse transcription polymerase chain reaction
